# HIV Env-Specific IgG Antibodies Induced by Vaccination of Neonatal Rhesus Macaques Persist and Can Be Augmented by a Late Booster Immunization in Infancy

**DOI:** 10.1128/mSphere.00162-20

**Published:** 2020-03-25

**Authors:** Alan D. Curtis, Maria Dennis, Joshua Eudailey, Korey L. Walter, Kenneth Cronin, S. Munir Alam, Neelima Choudhary, Ryan H. Tuck, Michael Hudgens, Pamela A. Kozlowski, Justin Pollara, Guido Ferrari, Koen K. A. Van Rompay, Sallie Permar, Kristina De Paris

**Affiliations:** a Department of Microbiology and Immunology, Center for AIDS Research, and Children’s Research Institute, School of Medicine, University of North Carolina at Chapel Hill, Chapel Hill, North Carolina, USA; b Duke Human Vaccine Institute, Duke University Medical Center, Durham, North Carolina, USA; c Department of Microbiology, Immunology, and Parasitology, Louisiana State University Health Sciences Center, New Orleans, Louisiana, USA; d Department of Biostatistics, Gillings School of Public Health, University of North Carolina at Chapel Hill, Chapel Hill, North Carolina, USA; e Department of Surgery, Duke University School of Medicine, Durham, North Carolina, USA; f Department of Molecular Genetics and Microbiology, Duke University Medical Center, Durham, North Carolina, USA; g California National Primate Research Center, University of California at Davis, Davis, California, USA; National Institute of Allergy and Infectious Diseases

**Keywords:** HIV, pediatric vaccination, memory antibody responses, adjuvant

## Abstract

The HIV epidemics in infants and adolescent women are linked. Young women of childbearing age are at high risk for HIV infection and, due to poor HIV testing rates and low adherence to antiretroviral therapy, are at high risk for mother-to-infant transmission. We hypothesize that HIV vaccine regimens initiated in early life would provide the necessary time frame to induce mature and highly functional Env-specific antibody responses that could potentially also protect against HIV acquisition later in life. The present study was designed to test two vaccine regimens, a clade C HIV Env protein vaccine (Env only) alone or combined with a modified vaccinia Ankara (MVA) vector expressing HIV Env (MVA/Env) for the induction and persistence of Env-specific antibody responses in an infant nonhuman primate model. Vaccination was initiated within the first week of life, with booster immunizations at weeks 6, 12, and 32. We demonstrate that both vaccine strategies were able to elicit durable Env-specific antibody responses that were enhanced by a late boost in infancy. Furthermore, we confirmed earlier data that intramuscular administration of the Env protein with the Toll-like receptor 7/8 (TLR7/8)-based adjuvant 3M-052 in stable emulsion (3M-052-SE) induced higher Env-specific antibody responses than vaccination with Env adjuvanted in Span85-Tween 80-squalene (STS) tested in a previous study. These results support the concept of early vaccination as a means to induce durable immune responses that may prevent HIV infection in adolescence at the onset of sexual debut.

**IMPORTANCE** The majority of new HIV-1 infections occur in young adults, with adolescent women being 3 times more likely to acquire HIV than young men. Implementation of HIV prevention strategies has been less successful in this age group; thus, a vaccine given prior to adolescence remains a high priority. We propose that instead of starting HIV vaccination during adolescence, an HIV vaccine regimen initiated in early infancy, aligned with the well-accepted pediatric vaccine schedule and followed with booster immunizations, will provide an alternative means to reduce HIV acquisition in adolescence. Importantly, the long window of time between the first infant vaccine dose and the adolescence vaccine dose will allow for the maturation of highly functional HIV Env-specific antibody responses. Our study provides evidence that early life vaccination induces durable Env-specific plasma IgG responses that can be boosted to further improve the quality of the antibody response.

## Abstract (precis)

The majority of new HIV-1 infections occur in young adults, with adolescent women being 3 times more likely to acquire HIV than young men. Implementation of HIV prevention strategies has been less successful in this age group; thus, a vaccine given prior to adolescence remains a high priority. We propose that instead of starting HIV vaccination during adolescence, an HIV vaccine regimen initiated in early infancy, aligned with the well-accepted pediatric vaccine schedule and followed with booster immunizations, will provide an alternative means to reduce HIV acquisition in adolescence. Importantly, the long window of time between the first infant vaccine dose and the adolescence vaccine dose will allow for the maturation of highly functional HIV Env-specific antibody responses. Our study provides evidence that early life vaccination induces durable Env-specific plasma IgG responses that can be boosted to further improve the quality of the antibody response.

## INTRODUCTION

Human immunodeficiency virus type 1 (HIV-1) prevention strategies such as pre- and postexposure prophylaxis (PrEP or PEP, respectively), and better access to HIV testing and counseling have significantly reduced global HIV infection rates. Nonetheless, in 2018, we faced 1.7 million new infections, with young adults representing a main risk group (1). Among adolescents, young women are 3 times more likely than young men to acquire HIV (2). Considering that these women exhibit low adherence to antiretroviral therapy (ART), are of childbearing age, and, if pregnant, are less likely to seek antenatal care, HIV infection in this age group also impedes the prevention of mother-to-child transmission of HIV with current standards of care. The fact that HIV infection in young women has remained relatively stable over the past decade underlines the importance of developing an HIV vaccine that can protect against sexual acquisition of HIV in adolescence (3). The stigma, the costs of lifelong ART in HIV-infected individuals, and the implementation and scale-up challenges of PrEP and PEP (4, 5) further enforce the need for a preadolescence HIV vaccine to stop the pandemic (6).

The induction of broadly neutralizing HIV Env-specific antibodies (bNAbs) and/or antibodies with Fc-mediated effector function is considered key to an effective HIV vaccine (7, 8). The development of highly functional antibodies is dependent on sufficient time for antibody maturation to increase avidity and enhance breadth (7, 8). As sexual maturity can be reached as early as 10 years of age, an HIV vaccine to protect against sexual transmission in adolescents, therefore, likely needs to be initiated in childhood. We propose, given the high acceptability of the pediatric vaccine schedule worldwide, it should be possible to align HIV vaccination with the Expanded Programme on Immunization (EPI) (9).

Most of the currently approved pediatric vaccines are initiated at 2 months of age or later to minimize potential interference with maternal antibodies and to overcome the challenges of the rather immature immune system at birth. Nonetheless, despite administration at birth, the hepatitis B vaccine has proven highly effective in providing protection against infection. Highly relevant to the studies here, the retrospective analysis of plasma samples from early HIV Env vaccine trials in human infants demonstrated that vaccination of neonates with HIV Env elicited persistent Env-specific plasma IgG antibodies (10). Furthermore, antibody responses were not only durable for up to 2 years, but the magnitude of plasma IgG antibodies specific to the V1V2 region exceeded those of adults in the RV144 trial (10, 11).

These data and findings from our earlier pediatric HIV Env vaccine strategies in the nonhuman primate model (12–14) provided the premise for our study described here. In those studies, aimed at developing a pediatric HIV vaccine to prevent breastmilk transmission of HIV, we documented that infant rhesus macaques can develop durable HIV Env-specific antibody responses (12). Env-specific antibody responses were enhanced by extending the intervals between immunizations and by applying adjuvants that act as agonists to Toll-like receptors instead of the commonly used aluminum hydroxide (alum) in pediatric vaccines (12, 13). Consistent with the data from early human pediatric vaccine trials (10), we also observed persistence of Env-specific antibody responses after vaccination in early life (12). In addition, we recently reported that passive administration of an HIV Env-specific broadly neutralizing monoclonal antibody at birth does not interfere with the induction of Env-specific antibody responses by vaccination in infant macaques (15).

**FIG 1:**
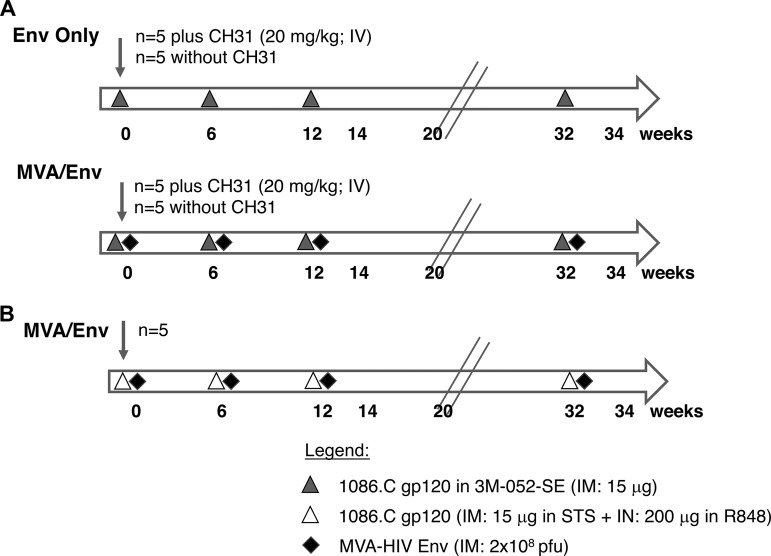
Experimental design and vaccine regimens. (A) Graphical outline of the vaccine schedule for the Env only (top) and MVA/Env (bottom) animal groups. Each vaccine group consisted of 10 infant rhesus macaques, with 5 animals per group also receiving the CD4 binding site broadly neutralizing antibody CH31 (15). Vaccine doses are listed in the legend, the time line of immunizations is listed below the time arrow in weeks of infant macaque age; all immunizations were given by the intramuscular route (i.m.). (B) The schedule of a previously published study (12) using a similar MVA/Env vaccine regimen. The Env protein was administered both by the intranasal (i.n.; 200 μg) and the i.m. (15 μg) routes, with R848 or STS as adjuvants for i.n. or i.m. immunizations, respectively.

Based on these results, we tested two vaccine regimens, an Env protein vaccine by itself or in combination with modified vaccinia Ankara (MVA) vaccine expressing HIV Env for the induction of durable antibody responses in infant macaques. The first vaccine dose was administered within the first week of life, followed by two boosts at weeks 6 and 12. To confirm the induction of memory B cells, we provided a late boost 20 weeks after the 3rd immunization (at week 32) and tested for recall responses. Thus, we initiate the vaccine during the neonatal period and gave the last booster immunization to 32-week-old infant macaques. Compared to human infants, dam-reared infant rhesus macaques start weaning from breastfeeding at approximately 6 months of age, whereas the nursery-reared infant macaques used in the present study were weaned off formula earlier and fully adjusted to chow by 6 months, and juvenile (adolescent) age is reached by 2 years (16). Our data support the idea that vaccination in early life elicits effector antibody responses that can be successfully boosted in infancy. In addition, we were able to further substantiate our earlier finding that the Toll-like receptor 7/8 (TLR7/8)-based adjuvant 3M-052 in stable emulsion (SE) is superior in inducing Env-specific antibody responses compared to a squalene-based adjuvant that was tested in a previous study (Fig. 1) (13).

## RESULTS

### Summary of vaccine-induced antibody responses after 3 immunizations.

The present study utilized a cohort of previously described infant rhesus macaques (RMs) (15) vaccinated with HIV Env protein (Env only) or MVA-Env plus Env protein (MVA/Env) in the presence or absence of the broadly neutralizing antibody CH31 (Fig. 1). CH31 was administered to provide passive immunity during the time antibody responses were developing in response to vaccination. The vaccine-induced antibody responses up to week 14, 2 weeks after the 3rd boost, were previously reported (15). The goal of the earlier study was to document that passive administration of a bNAb at the time of the vaccine prime does not interfere with the induction of vaccine-induced antibody responses in infants. Based on these results, we combined animals that had received the same vaccine regimen with or without administration of the bNAb CH31 for the present study (Table 1), followed them through week 32 to evaluate the persistence of antibody responses, and tested whether a final boost 20 weeks after the last immunization (at week 32) was able to further enhance these responses. The vaccine-induced antibody responses observed at week 14 served as comparative values for this analysis (Table 1).

**TABLE 1:** 1086.C Env-specific antibody responses after 3 immunizations (week 14)^*a*^

Response type	Antibody response (mean ± SD)		
	Without bnAb (*n* = 5)	With bnAb (*n* = 5)	Combined groups (*n* = 10)
Env Only			
Plasma IgG (ng/ml)	2.4 × 10^6^ ± 4.2 × 10^5^	1.8 × 10^6^ ± 8.3 × 10^5^	2.1 × 10^6^ ± 6.9 × 10^5^
Antibody avidity score	4.5 × 10^7^ ± 1.8 × 10^7^	1.2 × 10^8^ ± 1.0 × 10^8^	8.2 × 10^7^ ± 8.0 × 10^7^
CD4 blocking activity (%)	87.9 ± 5.8	91.1 ± 2.3	89.5 ± 4.5
ADCC endpoint titer	6.5 × 10^5^ ± 4.2 × 10^5^	7.3 × 10^5^ ± 5.2 × 10^5^	6.9 × 10^5^ ± 4.5 × 10^5^
MVA-Env/Env protein			
Plasma IgG (ng/ml)	1.4 × 10^6^ ± 6.6 × 10^5^	1.6 × 10^6^ ± 8.7 × 10^5^	1.5 × 10^6^ ± 7.4 × 10^5^
Antibody avidity score	4.8 × 10^7^ ± 3.0 × 10^7^	5.0 × 10^7^ ± 1.9 × 10^7^	4.9 × 10^7^ ± 2.4 × 10^7^
CD4 blocking activity (%)	90.7 ± 4.2	88.1 ± 4.2	89.4 ± 6.4
ADCC endpoint titer	5.4 × 10^5^ ± 3.2 × 10^5^	6.7 × 10^5^ ± 3.3 × 10^5^	6.1 × 10^5^ ± 3.1 × 10^5^

a Published by Dennis et al. (15).

### Persistence of plasma Env-specific IgG antibodies and boost of memory responses.

**FIG 2:**
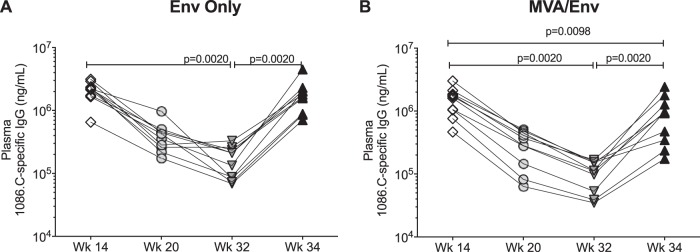
Plasma Env-specific IgG antibody concentrations. The concentrations of 1086.C-specific plasma IgG antibodies at weeks 14, 20, 32, and 34 are shown for Env-only- (A) or MVA/Env-vaccinated (B) infant rhesus macaques. Each symbol represents an individual animal. Within each vaccine group, Env-specific antibody concentrations between two time points were compared by Wilcoxon matched-pairs signed-rank test.

Compared to peak responses after the initial 3 immunizations, concentrations of 1086.C-specific plasma IgG from both Env-only and MVA/Env group infants decreased >10-fold from week 14 to week 32 (*P* = 0.002) (Fig. 2A and B). The late vaccine boost at week 32, 20 weeks after the last immunization, enhanced 1086.C-specific plasma IgG antibody concentrations in both vaccine groups compared to the trough point (*P* = 0.002). However, while mean 1086.C-specific plasma IgG antibody concentrations at week 34 in infants of the Env-only group were indistinguishable from week 14 levels (mean ± standard deviation [SD], 1.8 × 10^6^ ± 1.1 × 10^6^ versus 2.1 × 10^6^ ± 6.9 × 10^5^, respectively), the magnitude of average 1086.C-specific plasma IgG antibody concentrations in the MVA/Env group at week 34 stayed below that at week 14 (mean ± SD, 9.6 × 10^5^ ± 7.2 × 10^5^ versus 1.5 × 10^6^ ± 7.4 × 10^5^, respectively; *P* = 0.0098) (Fig. 2).

**FIG 3:**
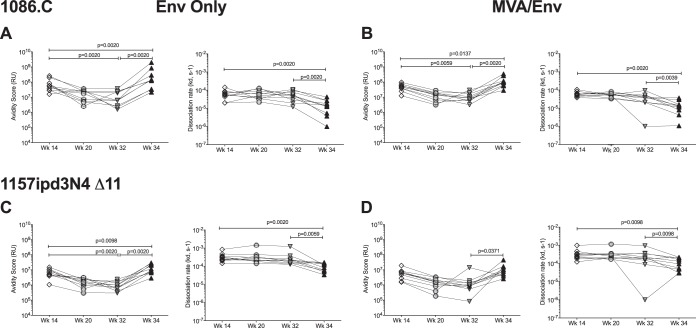
Avidity of Env-specific plasma IgG antibodies. The avidity of Env-specific antibodies was measured by SPR, and results are shown for the avidity binding score and the dissociation constant for antibodies against the vaccine immunogen 1086.C gp120 (A and B) and for clade C 1157ipd3N4Δ11 gp120 (C and D). Differences in avidity measurements between two distinct time points were determined by Wilcoxon matched-pairs signed rank test.

Consistent with the waning of 1086.C-specific plasma IgG antibodies over time, antibody avidity, measured by avidity score, was lower at week 32 than at week 14 in both vaccine groups (Fig. 3A). However, the dissociation constant remained stable over time, implying that the strength of antibody-antigen interaction was maintained (Fig. 3A). This conclusion is supported by other studies describing the dependence of the avidity score on antibody concentration and suggesting that avidity should be evaluated as a combined measure of avidity score and dissociation constant (17, 18). In response to the week 32 boost, an increase in the avidity of 1086.C-specific plasma IgG was observed in all vaccinated animals. Thus, from week 32 to week 34, avidity scores increased and dissociation rates decreased in both groups (Fig. 3A and B). Importantly, the postboost avidity of 1086.C-specific plasma IgG antibodies exceeded the avidity achieved after the 3rd immunization (see week 14 values in Table 1 and Fig. 3A and B). The ability to enhance antibody avidity by a late boost was further confirmed by measuring avidity scores and dissociation constants for IgG antibodies directed against clade C 1157ipd3N4Δ11 gp120 (Fig. 3C and D).

### Induction of 1086.C-specific antibodies in salivary secretions.

As HIV is primarily mucosally transmitted, we measured 1086.C-specific IgG and IgA antibodies in salivary secretions. At week 14, Env-specific antibodies were easily detected in both vaccine groups, but decreased subsequently (see Fig. S1A and B in the supplemental material). Although the week 32 booster immunization was able to restore the specific activity of Env-specific salivary IgG at week 34 to levels observed at week 14 in the Env-only group, the booster immunization was less effective in the MVA/Env group. These results mirrored our findings for plasma 1086.C-specific IgG (Fig. 2). In fact, in both vaccine groups, plasma 1086.C-specific IgG strongly correlated with the 1086.C IgG specific activity in saliva (Fig. S1C and D), suggesting that salivary IgG was transudated and not locally produced. A very similar pattern was observed for Env-specific IgA activity in saliva (Fig. S1E and F); Env-specific IgA antibodies were detected in all animals at week 14 but had sharply decreased by week 32 and were only minimally boosted. The source of Env-specific IgA antibodies in saliva samples could not be determined, because we did not have plasma IgA measurements.

10.1128/mSphere.00162-20.1 FIG S1 Env-specific antibody responses in saliva. Longitudinal data for the 1086.C gp120-specific IgG in saliva samples of animals in the Env only (A) and the MVA/Env (B) groups. Each symbol represents an individual animal. Within each vaccine group, Env-specific antibody concentrations between two time points were compared by Wilcoxon matched-pairs signed-rank test. (C and D) Correlation between plasma 1086.C-specific IgG and the specific IgG activity in saliva for both vaccine groups at week 34. Individual symbols represent individual animals. Data were analyzed by Spearman correlation. Note that similarly strong correlations were observed at week 14 (data not shown). Longitudinal data for the 1086.C gp129-specific IgA in saliva of Env only (E) and the MVA/Env (F) animals are documented. Each symbol represents an individual animal. Within each vaccine group, Env-specific antibody concentrations between two time points were compared by Wilcoxon matched-pairs signed-rank test. Download FIG S1, EPS file, 0.2 MB. Copyright © 2020 Curtis et al. 2020 Curtis et al. This content is distributed under the terms of the Creative Commons Attribution 4.0 International license.

### A late booster immunization in infancy enhances antiviral antibody functionality.

**FIG 4:**
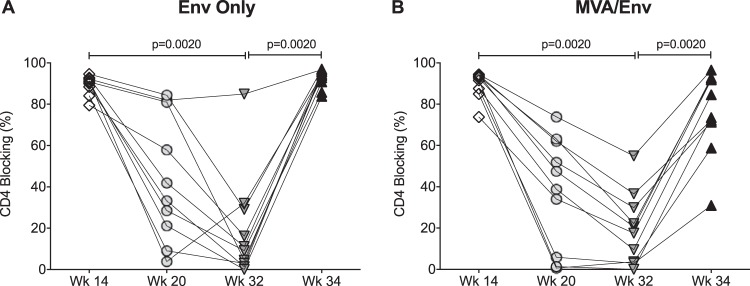
Vaccine-induced antibodies against the CD4 binding site. Longitudinal changes in the CD4 blocking function of Env-specific antibodies induced by the Env only (A) or MVA/Env (B) vaccine regimens are illustrated. Comparisons were performed applying the Wilcoxon matched-pairs signed-rank test.

The major goal of this study was to determine whether an additional booster immunization would improve not only the magnitude but also the function of vaccine-induced HIV Env-specific antibody responses. We first evaluated the ability of 1086.C-specific plasma IgG antibodies to recognize and block Env binding to the CD4 molecule. After the 3rd immunization of the original vaccine regimen, the CD4 blocking activity of Env-specific IgG ranged from 80% to 95% (Fig. 4A) and 74% to 95% (Fig. 4B) in infant RMs vaccinated with Env only and MVA/Env, respectively. In the following 20 weeks, the ability of Env-specific IgG to block the CD4 binding site decreased in all animals (Fig. 4). At week 32, the CD4 blocking function varied widely, ranging from 0% to 85% (mean ± SD, 19.1% ± 25.6%) in the Env-only group and from 0% to 60% (mean ± SD, 19.8% ± 17.2%) in MVA/Env-vaccinated infant RMs (Fig. 4). In the Env-only group, the week 32 boost was able to restore CD4 blocking function to week 14 levels, with a range of 84% to 97% (Fig. 4A). Although the average CD4 blocking activity at week 34 did not differ by Wilcoxon signed-rank test from that observed at week 14 in the MVA/Env-vaccinated infant RMs, 8 of 10 animals maintained reduced function compared to that at week 14, reflected in a wide range of CD4 blocking function (31% to 96%) (Fig. 4B).

**FIG 5:**
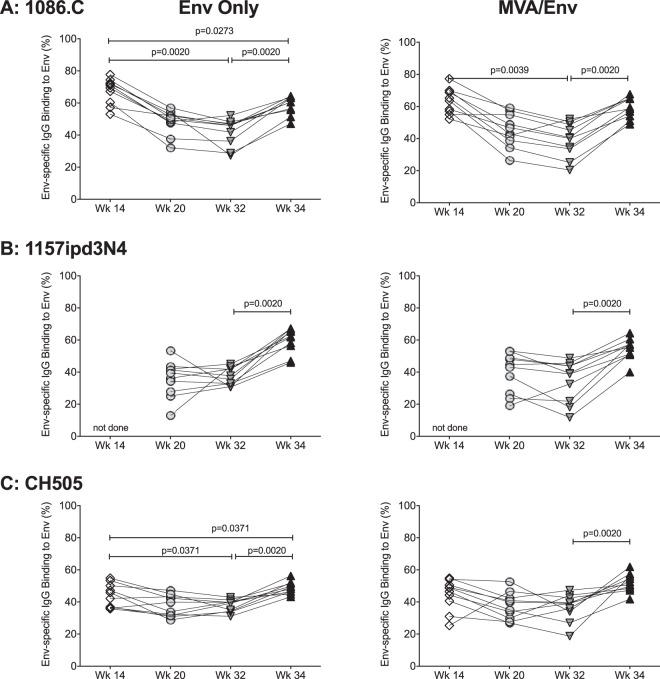
Ability of vaccine-induced Env-specific plasma IgG antibodies to bind to infected cells. Each panel shows on the *y* axes the percentage of Env only (left) or MVA/Env (right) vaccine-induced plasma IgG antibodies to bind to cells expressing 1086.C Env (A), 1157ipd3N4 Env (B), and CH505 Env (C) on their cell surface. The *x* axes report the time points of the study. Each line represents one animal, and symbols are harmonized with the other figures. Due to lack of plasma sample, we could not test for binding activity to 1157ipd3N4 Env at week 14. Differences in infected cell antibody binding between two distinct time points were determined by Wilcoxon matched-pairs signed-rank test.

We also assessed the ability of HIV Env-specific plasma IgG to bind to infected cells and to mediate antibody-dependent cytotoxicity (ADCC). Binding of infected cells was measured with an infected cell antibody-binding assay (ICABA), using cells infected with HIV-1 infectious molecular clone virus selected to match the vaccine immunogen 1086.C and two other clade C isolates, 1157ipd3N4 and CH505 (19, 20), to evaluate breadth. ADCC was assessed using target cells coated with Env gp120 representing the same virus isolates. We found that the binding of 1086.C-specific plasma IgG to cell surface-expressed 1086.C Env decreased from week 14 until week 32 in both vaccine groups (Fig. 5A) but increased after the boost at week 32. In MVA/Env-vaccinated infant RMs, the capacity of plasma IgG antibodies to bind to 1086.C Env expressed on the surface of infected cells was restored to week 14 levels (mean ± SD, 59.2% ± 6.5% versus 63.7% ± 8.0%, respectively), whereas antibody binding function in Env-only-vaccinated animals stayed slightly below levels observed at week 14 (mean ± SD, 58.7% ± 5.8% versus 67.5% ± 8.0%, respectively) (Fig. 5A). Although the limited plasma volumes that can be obtained from infant macaques prevented us from testing the week 14 time point for SHIV1157ipd3N4-binding antibodies, we were able to demonstrate that vaccine-induced Env-specific plasma IgG antibodies at week 32 were able to bind to cells expressing closely related clade C 1157ipd3N4 Env or CH505 Env, and, similarly to 1086.C binding, these responses increased after the boost (Fig. 5B and C).

**FIG 6:**
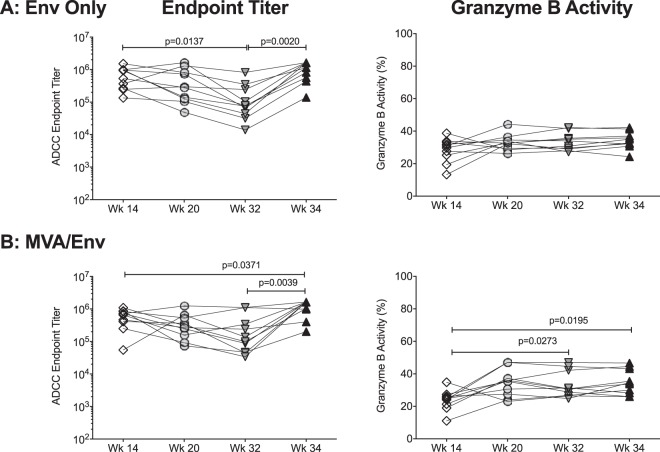
Persistence of ADCC function of 1086.C-specific plasma IgG antibodies. Graphs depict changes in ADCC endpoint titers (left) and peak granzyme B activity (right) from week 14 to week 34 in Env only- (A) or MVA/Env-vaccinated (B) infant RMs. Differences between two distinct time points were determined by Wilcoxon matched-pairs signed-rank test.

Mirroring the results of the ICABA, the ADCC endpoint titers decreased from week 14 to 32 in animals of the Env-only group but were restored to week 14 levels after the week 32 boost (Fig. 6A). Consistent with comparable average ADCC endpoint titers of Env-specific plasma IgG antibodies at weeks 14 and 34, no changes in granzyme B activity were observed in Env-only-vaccinated infant RMs (Fig. 6A). In contrast, in MVA/Env-vaccinated infant RMs, the week-14 ADCC endpoint titers were maintained until week 32, and peak granzyme B activity increased during this time period (Fig. 6B). After the late boost, ADCC endpoint titers and granzyme B activity surpassed those induced after 3 immunizations (week 14) (Fig. 6B).

**FIG 7:**
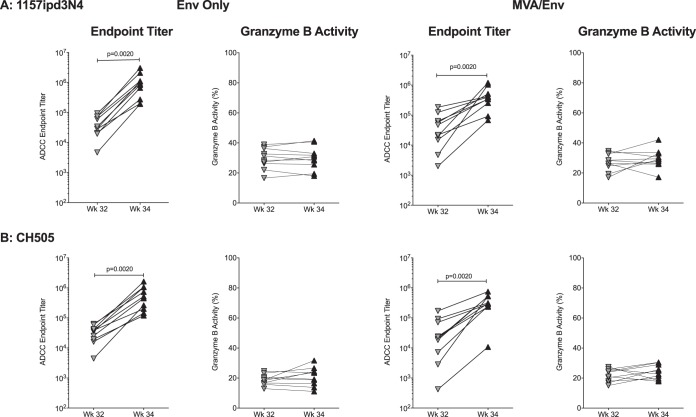
Vaccination with 1086.C gp120 induces antibodies with ADCC activity directed against other clade C gp120 molecules. Vaccination with 1086.C gp120 immunogens also induced plasma IgG antibodies with ADCC activity, measured as ADCC endpoint titers and peak granzyme B activity, that were directed against gp120 proteins of the clade C 1157ipd3N4 (A) or CH505 virus (B). Differences between two distinct time points were determined by Wilcoxon matched-pairs signed-rank test.

At week 32, ADCC function, although lower, was also detected against other clade C envelope molecules (Fig. 7). ADCC endpoint titers against 1157ipd3N4 or CH505 gp120 further increased in response to the week 32 boost, while peak granzyme B activity remained unchanged (Fig. 7A and B), a finding that was observed in both vaccine groups.

Finally, we also assessed the neutralizing capacity of Env-specific plasma antibodies. Our vaccine regimens were not designed to target the specific induction of neutralizing antibodies. Thus, consistent with our earlier studies (12, 15), we were able to detect tier 1 neutralizing antibodies against the clade C virus MW965 at week 14 (Fig. S1). As observed for most other antibody functions, the neutralizing activity declined over time but increased after the 4th immunization. Although median neutralizing 50% infective dose (ID_50_) titers exceeded those observed at week 14 (Env only: 8,407 versus 5,112; MVA/Env: 9,103 versus 7,294, respectively), the responses did not differ by Wilcoxon signed-rank test (see Fig. S2).

10.1128/mSphere.00162-20.2 FIG S2 Vaccine-induced tier 1 neutralizing antibodies. (A and B) Longitudinal data for plasma IgG antibodies that neutralize clade C MW965 infection in TZM-bl cells by 50%. Each symbol represents an individual animal. Within each vaccine group, Env-specific antibody concentrations between two time points were compared by Wilcoxon matched-pairs signed-rank test. Download FIG S2, EPS file, 0.1 MB. Copyright © 2020 Curtis et al. 2020 Curtis et al. This content is distributed under the terms of the Creative Commons Attribution 4.0 International license.

### Impact of adjuvant on vaccine-induced antibody responses.

In our efforts to optimize pediatric HIV vaccine strategies, we had previously compared different vaccine strategies, vaccine intervals, and various adjuvants with the goal to enhance Env-specific antibody responses (12, 13). As part of these studies, we had applied a similar MVA/Env regimen (12), but in the prior study, the 1086.C protein was given by the intranasal route (200 μg) adjuvanted with the TLR7/8 agonist R848 (25 μg) and by the intramuscular (i.m.) route (15 μg) adjuvanted with Span85-Tween 80-squalene (STS) (15 μg; STS/R848 vaccine group). In the previous study, the MVA/Env vaccine was also given at weeks 0, 6, and 12, with an additional boost at week 32. Therefore, we had the opportunity to confirm the superior adjuvant activity of 3M-052-SE in promoting pediatric antibody responses by comparing the Env-specific antibody responses elicited by the MVA-Env plus Env protein in STS/R848 in the prior study (12) to the ones elicited by the current vaccine regimen that included MVA-Env and the i.m. administration of 3M-052-SE adjuvanted 1086.C gp120. It should be noted that, due to limited plasma volumes available, we used historical data (12) for the comparison of plasma Env-specific IgG levels and ADCC function, whereas the ability of Env-specific IgG to block soluble CD4 and to bind to envelope protein on infected cells was tested with samples from both studies being run in parallel.

**FIG 8:**
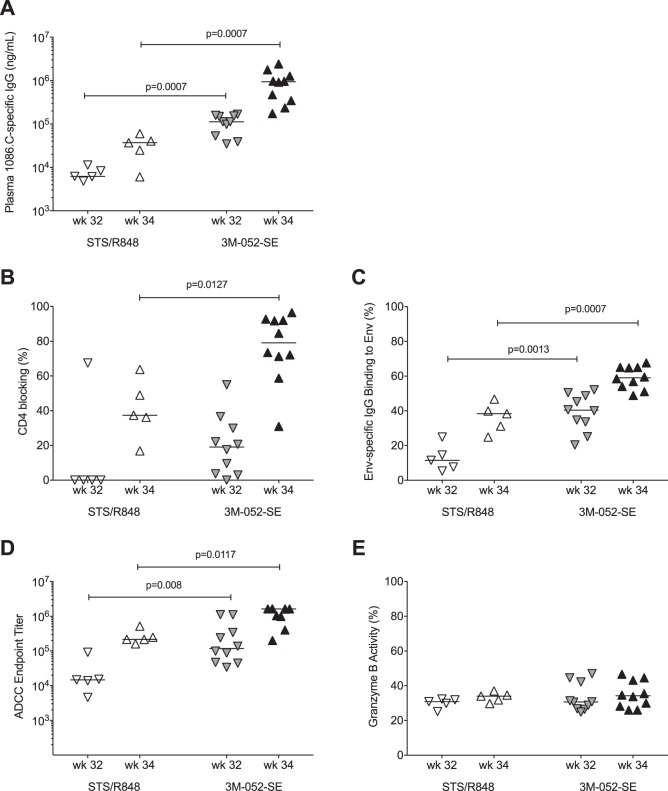
Comparison of 1086.C-specific IgG antibody responses induced by MVA/Env vaccination that utilized different adjuvants for Env protein administration. The magnitude (A), CD4 blocking activity (B), Env-specific binding on cells (C), ADCC endpoint titers (D), and peak granzyme B activity (E) of Env-specific plasma IgG antibodies at weeks 32 (time of boost) and 34 in animals receiving Env protein with STS/R848 adjuvants (empty symbols [12]) or 3M-052-SE adjuvant (filled symbols). Horizontal lines denote group median values. Antibody responses between the two different vaccine groups at the same time point were compared by Mann-Whitney test. Note that historical data for the STS/R848 regimen from our earlier study (12) were used for panels A, D, and E.

Prior to the boost at week 32, Env-specific IgG antibody concentrations were lower in infant RMs of the STS/R848 group than in MVA/Env-vaccinated animals in the present study (Fig. 8A), and this difference in Env-specific IgG antibody concentrations was maintained after the boost. Env-specific antibodies directed against the CD4 binding site were detected in only 1 of 5 infant RMs in the STS/R848 group at week 32, whereas 9 of 10 animals in the MVA/Env group of the present study had Env-specific antibodies that exhibited CD4 blocking activity (Fig. 8B). After the week 32 boost, animals in both groups and studies had detectable CD4-blocking Env-specific plasma IgG, but this activity was markedly higher in the infant RMs that received the Env protein adjuvanted with 3M-052-SE (Fig. 8B). Similarly, the ability of Env-specific plasma IgG antibodies to bind to 1086.C Env on infected cells prior to and postboost was higher in the 3M-052-SE group than in the STS/R848 group (Fig. 8C), and this finding was mirrored in endpoint ADCC titers (Fig. 8D). Although there was no difference in granzyme B activity between the groups (Fig. 8E), the higher endpoint titer in the 3M-052-SE group implies improved ADCC activity compared to that in the animals in the STS/R848 group of the previous study (12).

## DISCUSSION

Pediatric vaccination has enjoyed unparalleled success in preventing childhood disease and has drastically reduced infant and childhood morbidity and mortality. Despite intense efforts, an HIV vaccine has remained elusive. HIV Env-specific antibody responses are likely a key component in mediating protection against HIV acquisition. The induction of broadly neutralizing antibodies effective against the majority of circulating HIV strains is thought to depend on prolonged maturation of antibody responses to allow for the high level of somatic hypermutation observed in bNAbs isolated from HIV-infected adults (21). However, in infants, bNAbs appear to develop faster than in adults and exhibit less somatic hypermutation (22, 23), implying that the infant immune milieu might be advantageous to the development of broadly functional antibody responses against HIV. Therefore, we are aiming to test whether early life vaccination, with potential boosts during childhood, could be exploited to induce protective immunity against HIV acquisition prior to sexual debut in adolescence.

Toward this goal, here, we characterized longitudinal functional antibody persistence in a cohort of infant RMs vaccinated with HIV clade C Env protein or with Env protein plus MVA expressing the same Env protein. Our two vaccine regimens were not designed to elicit broadly neutralizing antibodies (24) but instead to elicit Env-specific antibodies with Fc-mediated effector function. Viral vectors in general (25), and poxvirus vectors such as MVA used in the present study or ALVAC used in the clinical HIV vaccine trials RV144 and HVTN702, have the advantage that they ensure stable antigen expression and exhibit self-adjuvanting activity (26, 27). Env protein-only vaccinations, however, generally elicit antibody responses with narrow specificity and relatively rapid antigen removal will likely limit antibody maturations, evident in lower somatic hypermutation rates (7). We included an Env-only arm in the present study because earlier studies in human infants had demonstrated that immunization of infants with MF59-adjuvanted HIV Env induced more durable antibody responses than adults receiving the same vaccine (10, 11); we did not aim to compare durability or magnitude of responses between the Env-only and MVA/Env regimens.

To understand long-term responses associated with HIV vaccination in our two cohorts, we followed the infants for 5 months after their first 3 immunizations. In both vaccine groups, vaccine-induced Env-specific plasma IgG persisted, although distinct functional antibody responses exhibited variable patterns of durability. The successful induction of vaccine-induced memory was confirmed, as several functional Env-specific responses were effectively recalled to peak levels after a 4th boost, 20 weeks after the 3rd immunization. Importantly, the avidity of Env-specific plasma IgG antibodies after the late boost exceeded levels observed at week 14 (Env only, 4.6-fold; MVA/Env, 2.6-fold). The importance of a late boost to maintain and increase immunity is also underlined by the finding that reduced HIV acquisition risk in human adults was of only transient nature in the RV144 trial (28). Correlates of protective immunity in the RV144 trial included Env-specific plasma IgG antibodies, especially those specific to the V1V2 epitope and mediating ADCC activity (29–31). Thus, it should be noted that ADCC endpoint titers and granzyme B activity remained relatively stable from week 14 to week 32 and were further enhanced after the 4th boost in the MVA/Env group.

These results confirm our earlier observation that the MVA/Env regimen can elicit durable antibody responses in infant macaques (12). The present study expanded on these findings by including a larger number of animals and by utilizing a more potent adjuvant for the Env protein. In the prior study, the 1086.C Env protein was administered both i.m. adjuvanted in STS and intranasally (i.n.) with the TLR7/8 agonist R848, whereas in the present study, the Env protein was given by the i.m. route only adjuvanted with 3M-052-SE. The vaccine regimen including the 3M-052-SE adjuvant resulted in the induction of higher magnitude and function of Env-specific antibodies, supporting the idea that age-appropriate adjuvants should be further explored in pediatric vaccine regimens that, for historical and safety reasons, primarily utilize alum adjuvants (13).

Our data from the Env-only vaccine regimen are consistent with the induction of persistent Env-specific antibody responses in human infants (10). It should be emphasized again that HIV Env-specific V1V2 IgG responses were induced at a higher magnitude in human infants than in adults (11). Similarly, the hepatitis B vaccine and the human papillomavirus vaccine are known to induce stronger antibody responses in infants than in adults (32, 33). Thus, the data of the present study support the idea that early life vaccination is an effective means to induce persistent HIV Env-specific antibody responses.

The present work is not without limitation, however. We followed infant macaques for only 34 weeks and for only 2 weeks after the final boost. Considering the transient protection observed with the ALVAC vaccine in the human RV144 trial and the recent stop of the HVTN702 trial, the durability of booster responses in the present study requires further testing. Furthermore, will additional booster immunizations result in affinity maturation and higher somatic hypermutation rates in Env-specific antibodies? The exploration of these questions was beyond the scope of the present study, but additional studies are under way to define somatic hypermutation rates in Env-specific B cells (S. Berendam, K. De Paris, S. Permar, and G. G. Fouda, unpublished data). The increase in Env-specific IgG avidity after the 4th boost compared to that at week 14 suggests that even nonpersistent viral vectors can drive functional maturation of antibody responses. Greater periods between extended boosts and potentially different Env immunogens to increase breadth need to be investigated and optimized (34) if we are to successfully carry immunity through adolescence. The effect of continued booster immunizations through adolescence and protection against antigenic challenge represent major unanswered questions in the field of pediatric vaccination. The requirement for frequent vaccinations may also present a hurdle to vaccine implementation in resource-limited settings (35). Initiation of an HIV vaccine series in early life could potentially be tailored to be aligned with the Expanded Programme on Immunization (9) to avoid challenges associated with immunizations starting in adolescence (36). The work presented here gives credence to the idea that an HIV vaccine started in early life could provide durable immune responses that may provide protection against HIV to adolescents at sexual debut and beyond.

## MATERIALS AND METHODS

### Animals.

The 20 simian immunodeficiency virus (SIV)- and type D retrovirus-negative newborn rhesus macaques (Macaca mulatta), both male and female (Table 1), were part of a previous published study (15). Infant macaques were nursery reared at the California National Primate Research Center (Davis, CA) in accordance with the Guide for Care and Use of Laboratory Animals outlined by the American Association for Assessment and Accreditation of Laboratory Animal Care (37). The International Guiding Principles of Biomedical Research Involving Animals were strictly adhered to (38). The UC Davis Institutional Animal Care and Use Committee approved all methods. Ketamine anesthesia (10 mg/kg; Parke-Davis, Morris Plains, NJ) was administered i.m. prior to all procedures.

### Vaccine strategy.

Infants and their vaccine regimen in the present study were previously described (Fig. 1) (15). For practical purposes, to synchronize the time of vaccination, animals were between 4 and 10 days old at the time of their first immunization (see Table S1 in the supplemental material). Vaccine groups were defined by the HIV Env immunogen. Infant macaques in the Env-only group (*n* = 10) received 15 μg HIV-1 Env 1086.C gp120 protein plus 250 μl 3M-052 adjuvant in stable emulsion (13) in a total volume of 500 μl administered intramuscularly (i.m.) divided over both quadriceps at weeks 0, 6, and 12. Animals designated MVA/Env (*n* = 10) were immunized with the same HIV-1 Env 1086.C gp120 protein vaccine but, in addition, received 10^8^ PFU of an MVA-HIV-Env construct (250 μl divided across both biceps). The MVA-HIV Env construct expressing 1086.C gp120 was generated as previously described (12). For the purpose of the present study, we disregarded that one-half of the animals in the Env-only and MVA/Env groups (*n* = 5/group) received a single dose (20 mg/kg; intravenously) of human CH31 IgG at week 0 (Fig. 1). As we could demonstrate that the CH31 antibody was rapidly depleted and did not interfere with HIV Env vaccine-induced antibody responses (15), animals receiving the same vaccine regimen with or without the CH31 Ab were combined in the present study (Table 1). In the study described here, all animals were boosted a final time at week 32 with the same vaccine regimen give at weeks 6 and 12 (Fig. 1) to test for vaccine-induced memory responses.

10.1128/mSphere.00162-20.3 TABLE S1 Study animals. Download Table S1, PDF file, 0.1 MB. Copyright © 2020 Curtis et al. 2020 Curtis et al. This content is distributed under the terms of the Creative Commons Attribution 4.0 International license.

### Sample collection and processing.

Specimen preparation from animals in the present study was recently described as part of a separate study (15). In addition to previous sample collection, we isolated peripheral blood mononuclear cells (PBMC) and plasma from EDTA-anticoagulated whole blood at weeks 20 and 32 and at necropsy at week 34.

### Measurement of HIV Env-specific antibodies by ELISA.

The plasma concentrations of HIV Env-specific antibodies were determined by enzyme-linked immunosorbent assay (ELISA) (12); 384-well plates were coated with 3 μg/ml of 1086D7 gp120K160N overnight at 4°C and then blocked with superblock (phosphate-buffered saline [PBS] plus 4% whey, 15% normal goat serum, and 0.5% Tween 20). After washing, serially diluted plasma was added to the plate. IgG antibodies were detected with peroxidase-labeled anti-monkey IgG (Southern Biotech), followed by tetramethylbenzidine (TMB; KPL) and stop solution. Immediately after addition of the stop solution, absorbance was read at 450 nm. B12R1 was used as a standard (39). The concentration of HIV Env-specific IgG was calculated using a five-parameter fit curve relative to the standard using SoftMax Pro 6.3 software (Molecular Devices). To account for nonspecific binding, the positivity cutoff was selected as the concentration corresponding to 3 times the optical density (OD) of blank wells.

### HIV Env-specific antibodies in saliva samples.

The specific activity of 1086.C gp120 -specific IgG and IgA was measured as previously described (12).

### Antibody avidity.

The avidity of anti-C.1086 gp120, anti-SHIV1157, and anti-SHIVCH505 plasma IgG antibodies was measured using surface plasmon resonance (SPR) as described (12). Nonspecific binding of a preimmune (time zero) sample was subtracted from each postimmunization IgG sample binding data. Data analyses were performed with BIAevaluation 4000 and BIAevaluation 4.1 software (BIAcore/GE Healthcare). Binding responses were measured by averaging postinjection response unit (RU) over a 10-s window, and dissociation rate constant, *K_d_* (s^−1^), was measured during postinjection phase (after stabilization of signal). A positive response was defined by an RU value of ≥10. The relative avidity binding score was calculated as follows: avidity score (RUs) = (binding response units/*K_d_*).

### Soluble CD4 blocking antibody assay.

CD4 blocking assays were performed using ELISA as previously described (40). Briefly, plates were coated with B.JRFL gp120 overnight at 4°C and blocked with PBS containing 4% (wt/vol) whey protein, 15% normal goat serum, 0.5% Tween 20, and 0.05% sodium azide for 1 h at room temperature. Rhesus macaque plasma was diluted 1:50 and plated in triplicates into the wells. Ten microliters of a predetermined saturating concentration of sCD4 (Progenics Pharm Inc., 0.64 μg/ml) was added following the plasma incubation step. Soluble CD4 binding was determined using biotinylated anti-CD4 monoclonal antibody (MAb) OKT4 (0.015 μg/ml) and streptavidin-horseradish peroxidase (HRP) at 1:30,000 dilution followed by TMB substrate. Plates were subsequently read with a plate reader at 450 nm. Percent inhibition was calculated as follows: 100 − (plasma triplicate mean/no plasma control mean) × 100.

### Infected cells antibody binding.

Plasma antibody binding to HIV-1 Env expressed on the surfaces of infected cells was measured using an infected cell binding assay as previously described (19, 20). In short, mock- or HIV-1 infectious molecular clone (IMC) virus-infected CEM.NKR_CCR5_ cells were infected for 48 to 72 h. Cells were subsequently cultivated in the presence of diluted plasma samples, stained with a viability marker, anti-CD4 antibody (clone OKT4; eBioscience, Waltham, MA), fixed, and permeabilized prior to staining with RD1-conjugated anti-p24 MAb KC57 (Beckman Coulter, Inc., Indianapolis, IN) and fluorescein isothiocyanate (FITC)-conjugated goat anti-rhesus IgG(H+L) polyclonal antiserum (Southern Biotech, Birmingham, AL). At time of assay, C.1086-infected cells were 90% p24^+^, and 68% of the infected cells had downregulated cell surface expression of CD4; SHIV1157ipd34N-infected cells were 64% p24^+^, and 50% of the infected cells had downregulated CD4; and CH505-infected cells were 32% p24^+^, and 58% of the infected cells had downregulated CD4. IgG binding was identified as viable p24^+^ FITC^+^ events. Data represent the frequency of cells positive for IgG binding to Env for postvaccination samples compared to that in the prevaccination sample. Values were normalized by subtraction of the frequency of positive cells observed for cells stained with secondary antibody alone and mock-infected cells.

### Antibody-dependent cellular cytotoxicity.

ADCC activity against CCR5^+^ CEM.NKR T cells (AIDS Reagent Program) coated with the C.1086 gp120, clade C SHIV1157ipd3N4 gp120, or C SHIVCH505 gp120 was measured by the GranToxiLux (GTL) assay as described (12, 41). Briefly, 4-fold serial plasma dilutions beginning at 1:100 were applied to cryopreserved human PBMCs from an HIV-seronegative donor with the 158F/V genotype for FcγRIIIa (42). Data were reported as the percentage of cells possessing active granzyme B relative to all viable target cells after background activity (effector and target cells in the absence of plasma) was subtracted. Endpoint titers were calculated by interpolating the dilutions of plasma which intercept the positive cutoff.

### Tier 1 neutralizing antibody assay.

Neutralization of tier 1 and tier 2 clade C viruses by plasma was measured in TZM-bl cells by reduction in luciferase reporter gene expression after a single round of infection, as described (43). Neutralization was assessed using MW965.LucR.T2A.ecto/293T (tier 1) and C.1086_B2.LucR.T2A.ecto/293T (tier 2) infectious molecular clones (IMCs). The broadly neutralizing antibodies b12R1 or VRC01 were used as positive controls. The 50% inhibitory dose (ID_50_) was calculated as the plasma dilution or antibody concentration that caused a 50% reduction in relative light units (RLU) compared to that in the virus control wells after subtraction of cell control RLU. The preimmunization time point (week 0) was used to measure background levels of neutralization. None of the vaccine regimens tested elicited tier 2 neutralizing antibody responses; therefore, only tier 1 neutralizing antibodies are reported (Fig. 1).

### Statistical analysis.

All reported *P* values are exact, and all calculations were performed with GraphPad Prism, version 6.0 for Mac OS X (GraphPad Software, La Jolla, CA). Antibody responses of the same animals within a vaccine group at different time points were analyzed using the Wilcoxon matched-pairs signed-rank test. Comparisons of antibody responses between different vaccine regimens (see Fig. 8) were conducted using the Mann-Whitney test. Plasma and salivary antibody levels were compared by Spearman correlation.
