# Supplementary material for: HIV Env-Specific IgG Antibodies Induced by Vaccination of Neonatal Rhesus Macaques Persist and Can Be Augmented by a Late Booster Immunization in Infancy
Source: mSphere. 2020 Mar 25;5(2):e00162-20. doi: 10.1128/mSphere.00162-20 (PMC7096624; doi:10.1128/mSphere.00162-20)
Supplement: TABLE S1 [file mSphere.00162-20-st001.pdf]

Supplemental Table 1: Study Animals

Vaccine	Animal ID	Sex	Age at 1st Immunization (days)
**Env**	RM1	male	9
	RM2	male	8
	RM3	male	7
	RM4	female	5
	RM5	female	5
	RM6	male	10
	RM7	male	6
	RM8	male	6
	RM9	female	5
	RM10	male	4
**MVA/Env**	RM11	male	9
	RM12	male	8
	RM13	female	7
	RM14	female	6
	RM15	female	5
	RM16	male	8
	RM17	male	6
	RM18	female	5
	RM19	female	5
	RM20	male	4
